# The molecular mechanism of METTL3 promoting the malignant progression of lung cancer

**DOI:** 10.1186/s12935-022-02539-5

**Published:** 2022-03-24

**Authors:** Chao Ma, Rui-Jie Ma, Kang Hu, Qi-Ming Zheng, Ye-Peng Wang, Nan Zhang, Zhi-Gang Sun

**Affiliations:** 1 Department of Thoracic Surgery, Central Hospital Affiliated to Shandong First Medical University, 105 Jiefang Road, Jinan, 250013 Shandong China; 2 School of Clinical Medicine, Weifang Medical University, Weifang, 261053 Shangdong China; 3 Cheeloo College of Medicine, Shandong University, Jinan, 250013 Shangdong China; 4 Breast Center, Central Hospital Affiliated to Shandong First Medical University, 105 Jiefang Road, Jinan, 250013 Shandong China

**Keywords:** METTL3, m^6^A, Lung cancer, Malignant progression, Prognosis, Tumor microenvironment, Inhibitors

## Abstract

Lung cancer remains one of the major causes of cancer-related death globally. Recent studies have shown that aberrant m^6^A levels caused by METTL3 are involved in the malignant progression of various tumors, including lung cancer. The m^6^A modification, the most abundant RNA chemical modification, regulates RNA stabilization, splicing, translation, decay, and nuclear export. The methyltransferase complex plays a key role in the occurrence and development of many tumors by installing m^6^A modification. In this complex, METTL3 is the first identified methyltransferase, which is also the major catalytic enzyme. Recent findings have revealed that METTL3 is remarkably associated with different aspects of lung cancer progression, influencing the prognosis of patients. In this review, we will focus on the underlying mechanism of METT3 in lung cancer and predict the future work and potential clinical application of targeting METTL3 for lung cancer therapy.

## Introduction

Lung cancer is one of the most common malignant tumors with the highest mortality rate worldwide [1–3]. According to histological appearance, lung cancer is classified into small cell lung cancer (SCLC) and non-small cell lung cancer (NSCLC) [4, 5]. Unfortunately, due to the lack of effective means of early diagnosis, most patients with lung cancer are found to be in an advanced stage and have a poor prognosis [6]. Even with the development of multidisciplinary comprehensive management of lung cancer, the overall survival (OS) of patients with lung cancer is still very low, about 15% [7].

As we all know, tumorigenesis is an extremely complex biological process involving genomics and epigenetics [8, 9]. There is strong evidence that epigenetic modification has a profound impact on the occurrence and development of tumors without DNA sequence changes [10]. The epigenetic modifications include DNA methylation, histone modification, RNA modification, and noncoding RNA [11, 12]. However, with the studies of DNA methylation, histone modification and noncoding RNA in full swing, we know little about RNA modification.

At present, more than 170 RNA modifications have been found in mammals [13], while m^6^A modification is the most abundant RNA chemical modification, accounting for approximately 60% of all RNA modifications [14, 15]. RNA m^6^A modification was reported as early as 1970 [16, 17], but it was not until the emergence of methylated RNA immunoprecipitation sequencing (MeRIP-seq) that m^6^A modification became the focus of epigenetic modification. In addition, aberrant m^6^A levels mediated by METTL3 have been reported to be involved in the malignant progression of various tumors, including proliferation, invasion, metastasis, and drug resistance.

**Fig. 1:**
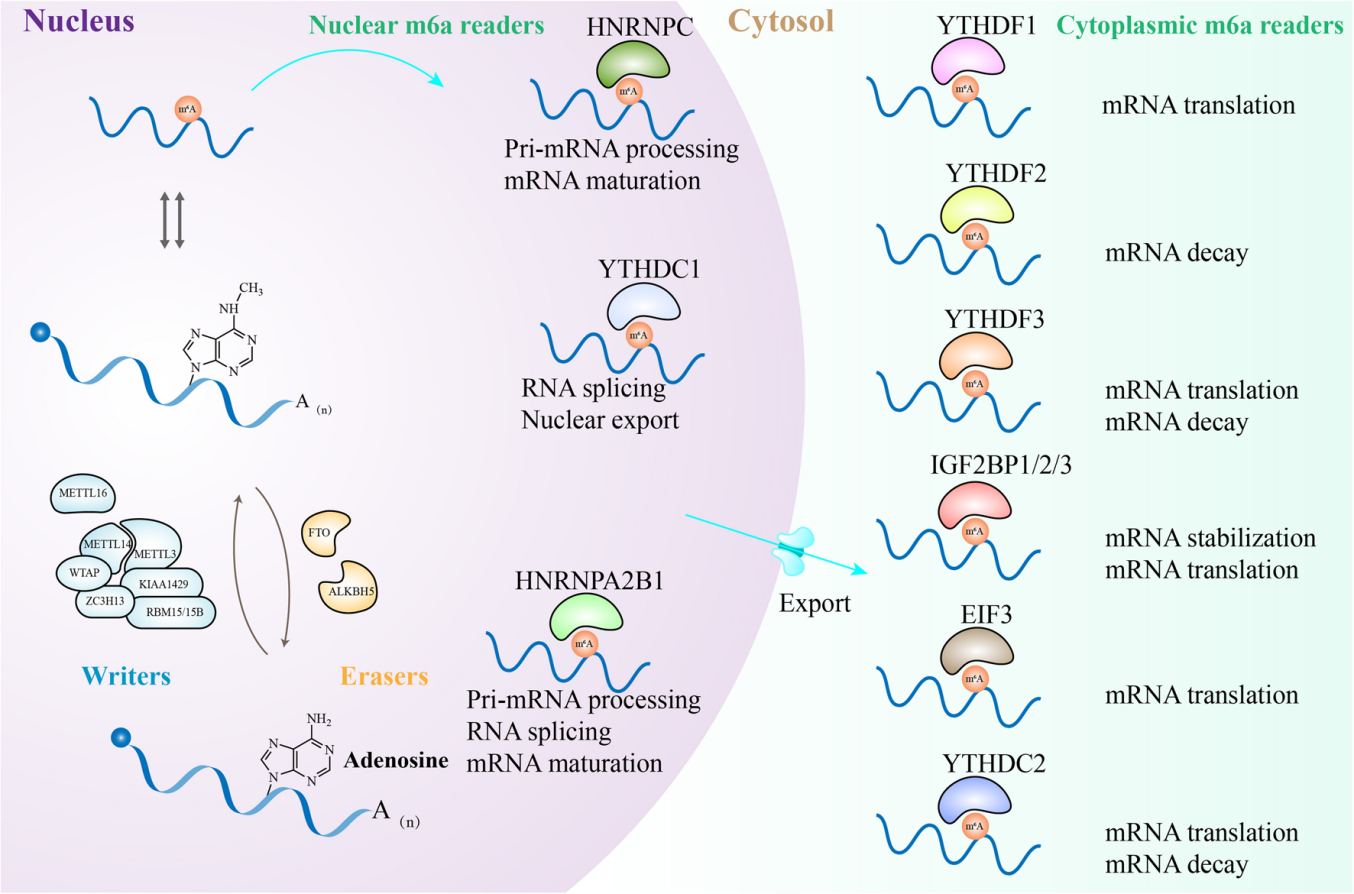
Molecular components involved in the dynamic and reversible process of m^6^A methylation include methyltransferases (METTL3, METTL14, METTL16, WTAP, KIAA1429, ZC3H13, RBM15/15B), demethylases (FTO, ALKBH5) and RNA-binding proteins (YTHDF1, YTHDF2, YTHDF3, YTHDC1, YTHDC2, IGF2BP1/2/3, HNRNPC, HNRNPA2B1). METTL3 has an influence on the expression of key oncogenes by regulating RNA metabolism, such as Pri-mRNA processing, RNA splicing, maturation, stability, translation, decay, and nuclear export

However, RNA m^6^A modifications account for about 0.1–0.4% of the isolated RNA from eukaryotic cells [16]. Studies have shown that the sites modified by m^6^A are highly conservative and tend to be present in the consensus sequence Pu[G > A] m^6^AC[U > A > C](Pu, purine) [18]. Of note, m^6^A sites are mainly enriched near the 3' untranslated region (UTR), stop codon and long internal exon [18, 19], and play an important role in regulating RNA metabolism. In mammals, m^6^A modification is dynamically reversible, installed by the methyltransferase complex (writers) and removed by demethylases (erasers) [20, 21]. At least eight methyltransferases are found in the methyltransferase complex, including METTL3, METTL14, WTAP, KIAA1429(VIRMA), RBM15, HAK AI, ZC3H13(KIA A0853), and METTL16. In the methyltransferase complex, METTL3 is the first identified and the sole catalytic subunit that can catalyze the transfer of a methyl group from *S*-adenosylmethionine (SAM) to the N6-position of adenosine [13]. In addition, RNA binding proteins, also called readers, can selectively recognize and bind to the m^6^A modification sites of target RNAs, thereby participating in the RNA metabolism process, including RNA splicing, maturation, decay, translation, stabilization, Pri-mRNA processing, nuclear export [22, 23] (Fig. 1). With the deepening of the research, more and more novel m^6^A-related regulators will be identified.

Recent findings have revealed that METTL3 is closely related to the progression of a variety of tumors, including lung cancer. In the present review, we will discuss the underlying molecular mechanism of METTL3 in the occurrence and development of lung cancer and predict the future research direction, as well as the potential clinical application of targeting METTL3 in lung cancer treatment.

## Effects of methyltransferase METTL3 on lung cancer

Accumulating evidence has shown that abnormal m^6^A levels mediated by METTL3 are involved in the malignant progression of lung cancer, including cell proliferation, invasion, metastasis, angiogenesis, drug resistance, glycolysis, cancer stem cells, and tumor environment [24–26]. Therefore, we summarize the recent findings of METTL3 in the tumorigenesis of lung cancer (Table 1).

**Table 1:** Effect of METTL3 on lung cancer

Role	Cell line	Animal mode	Up-regulator	Target	Mechanism	Biological function	Refs
Oncogene	A549, H1299, H1792, BJ, IMR-90	Female NU/J (Nude) immunodeficient mice	–	EGFR and TAZ	Enhance oncogene translation	Promote cell growth invasion and survival	[42]
	A549 NCI-H460	–	miR-33a	EGFR and TAZ	Downregulate the expression of METTL3 and downstream genes	Inhibit cell proliferation	[32]
	A549, H1299 HEK293T BJ, NIH-3T3, HeLa cells, MEFs	–	–	EIF3h	Enhance translation of oncogenic mRNAs	Promote oncogenesis	[43]
	HBEC, A549, H1299, Calu6, H520, 95-D	–	–	MALAT1-miR-1914-3p-YAP	Promote YAP translation and increase YAP activity	Promote tumor drug resistance and metastasis	[38]
	–	BALB/c nude mice	–	miR-143-3p/VASH1 axis	Activate miR-143-3p/VASH1 axis	Promote the brain metastasis of lung cancer	[59]
	A549, H1299	–	miR-600	EGFR, TAZ, DNMT3a	Activate PI3K pathway and upregulate expression of apoptosis-related proteins	Promote cell proliferation, migration, invasion of lung cancer cells and induce cell apoptosis,	[102]
	A549	–	–	EZH2	Induce m^6^A modification on EZH2 mRNA	Promote cell EMT, migration, invasion	[83]
	H1299, A549, EBC-1, HCC827, Calu-3, H661, H596, H358, H460, H1650, H1975, H1395, and H292	–	–	c-Met	Increase c-Met mRNA methylation	Induce drug resistance	[91]
	A549, HCC827, PC9	–	H_2_S	PRPF6	Promote PRPF6 gene spicing and translation	Promote cell growth, proliferation, invasion	[103]
	A549, LC2/ad	–	–	JUNB	Increase the stability of JUNB mRNA	Induce EMT	[35]
	HBE	BALB/c nude mice	–	ZBTB4	Attenuate the mRNA stability of ZBTB4	Induce EMT	[41]
	PC9, H3255	–	–	c-Met	Regulate PI3k/AKT pathway	Promote drug resistance	[51]
	A549, H1299, H520, H1975	BALB/c nude mice	–	miR-1246	Upregulate the expression of miR-1246 and downregulate PEG3	Promote lung cancer occurrence and progression	[104]
	H1650, A549, BEAS-2B	BALB/C nude mice	CircPUM1	–	Upregulate the expression of METTL3 via targeting mir-590-5p	Promote cell proliferation and glycolysis in NSCLC	[31]
	BEAS‐2B, NCI-H1299, A549, HCC827, and NCI‐H1650	Male mice	–	LncABHD11‐AS1	Enhance LncABHD11-AS1 stability	Promote the proliferation and Warburg effect of NSCLC cells	[30]
	A549, PC9, H1299, H1975 and HCC827, BEAS‑2B	BALB/c nude mice	–	Bcl-2	Enhance the expression of Bcl-2	Promote cell growth, survival, migration in NSCLC	[81]
	H1299, H460, and A549, BEAS-2B	–	miR-338-5p	C-myc	Regulate the expression of C-myc	Promote cell growth and migration	[105]
Tumor Suppressor	HEK-293T, BEAS-2B, A549, NCI-H1299, PC-9, NCI-H1975, NCI-H441, NCIH1650, HCC827, NCI-H292, Calu-1	KP mice, Athymic nude mice	–	YTHDC2	Promote the degradation of SLC7A11 mRNA	Suppress tumorigenesis	[34]
	HEK293T, 16HBE, PGCL3, H460, H1299, and A549	BALB/c nude mice	miR-4443	FSP1	Inhibit FSP1 m^6^A modification-mediated ferroptosis	Inhibit Cisplatin resistance	[52]
	HCC827, PC9	BALB/c nude mice	–	FBXW7	Enhance FBXW7 mRNA translation	Suppress cell proliferation	[106]

## Effects of METTL3 on the proliferation of lung cancer

Lung cancer is a malignant tumor originating from bronchial mucosal epithelial cells that is closely associated with excessive cell division, cycle disturbance, and apoptosis dysregulation [27–29]. It has been reported that long noncoding RNA (LncRNA) ABHD11-AS1 was highly expressed in NSCLC, closely related to poor prognosis. Mechanistically, METTL3 increases the transcript stability of ABHD11-AS1 and reduces the expression of its downstream gene KIF4, promoting cell proliferation [30]. In contrast, RNA pumilio RNA binding family member 1 (circPUM1), a functional circRNA, promotes NSCLC cell growth by activating the miR-590-5p/METTL3 axis [31]. Thus, METTL3 participates in the initiation and development of lung cancer by modifying noncoding RNA. Interestingly, miR-33a can inhibit NSCLC cell proliferation by downregulating METTL3 expression and its downstream genes, such as epidermal growth factor receptor (EGFR) and transcriptional coactivator with PDZ-binding motif (TAZ), via directly targeting the 3'UTR of METL3 mRNA [32].

In addition, METTL3 can also act as a tumor suppressor in lung cancer. Wu et al. found that numerous m^6^A methylation sites on FBXW7 mRNA in lung adenocarcinoma (LUAD) by using MeRIP-qPCR analysis. Further study showed that METTL3 upregulates FBXW7 expression to regulate proliferation or apoptosis-related genes such as Bax, c-Myc, Mcl-1, in an m^6^A manner [33]. Similarly, METTL3 represses LUAD tumorigenesis by regulating SLC7A11 mRNA expression. Mechanistically, YTHDC2 (reader) preferentially binds to m6A-modified SLC7A11 mRNA, which makes it more likely to be degraded, preventing cystine uptake and antioxidant function [34].

## Effects of METTL3 on the invasion, migration and metastasis of lung cancer

Accumulating studies have shown that METTL3 overexpression in lung cancer tissues was significantly higher than that in normal cells and was strongly associated with tumor invasion, migration, and metastasis [35–37]. In NSCLC, METTL3-mediated Yes-associated protein (YAP) overexpression leads to tumor metastasis. By analyzing the upstream regulatory mechanism of YAP, researchers found that METTL3 not only upregulates the m^6^A level of YAP transcript to enhance its translation but also activates the MALAT1-miR-1914-3p-YAP axis to increase the stability of YAP transcript [38]. In lung cancer epithelial-mesenchymal transition (EMT) models mediated by TGF-β, METTL3 markedly accelerates the EMT process [35]. Mechanistically, METTL3 significantly enhances JUNB mRNA stability dependent of m^6^A methylation. In contrast, E-cadherin expression was upregulated when METTL3 knockdown was performed.

Moreover, CTNNB1 gene  encodes β-catenin protein. The m^6^A level of CTNNB1 mRNA is abnormally increased, closely related to the EMT process in HeLa cell line. Mechanistically, METTL3 modifies the 5'UTR region of CTNNB1 mRNA and negatively regulates CTNNB1 mRNA stability and translation [39]. In addition, METTL3 upregulates the expression of transcription factor E2F1, thereby indirectly downregulating β-catenin protein. Intriguingly, this study also revealed that YTHDF1 selectively binds eIF4E1 or eIF4E3 to regulate β-catenin expression in a noncanonical pathway, according to the level of METTL3 in the cell. For example, in METTL3 knockdown cells, YTHDF1 tends to bind the oncoprotein eIF4E1 to upregulate β-catenin.  Eventually, METTL3 can also decrease c-Met kinase expression to repress membrane localization of β-catenin, inhibiting cell migration.

The m^6^A levels were reported to be affected by environmental factors, including particulate matter (PM_2.5_) [40] and cigarette smoke [41]. Recent studies have shown that METTL3 overexpression induced by smoke exposure downregulates E-cadherin to accelerate the malignant transformation of normal lung tissue in mice by activating the ZBTB4/EZH2/ H3K27me3 axis [41]. It has been reported that METTL3 participates in m^6^A-modified mRNA translation independently of its catalytic activity or m^6^A readers in lung cancer lines [42]. In METTL3 mutation assays, its N-terminal domain (1–200 amino acids) did not exhibit methyltransferase catalytic activity but is sufficient to increase the translation efficiency of target mRNAs. Mechanistically, METTL3 interacts with eIF3h to form the RNA looping to enhance significantly the translation efficiency of polyribosomes, leading to an increase in the expression of key oncogenes such as EGFR and TAZ [43]. However, METTL3 approximately selectively binds only 22% of RNA containing the m^6^A methylation site [44], so it is vital to explore selection mechanisms of METTL3 for target mRNAs to elucidate its biological function.

## Effects of METTL3 on drug resistance of lung cancer

Drug resistance is the major reason for the failure of most solid tumor treatments [8, 45, 46], its mechanism is quite complicated, including cancer stemness, the ABC transporter family, noncoding RNA regulation, tumor microenvironment, hypoxia, autophagy, DNA damage and repair, and epigenetic modification. However, there is a lot of evidence that METTL3 is linked to drug resistance in many different types of tumors, including lung cancer [27].

In a murine cisplatin-resistant lung cancer model, METTL3 upregulates YAP expression to promote drug resistance and metastasis by YTHDF1/3, eIF3b and the MALAT1-miR-1914-3p-YAP axis [38]. In contrast, METTL3 knockdown increased the sensitivity of lung cancer cells to Cisplatin by downregulating YAP expression. C-Met overexpression in NSCLC was positively correlated with Crizotinib resistance [47, 48]. NSCLC cells treated with Chidamide increased sensitivity to Crizotinib in vivo and vitro. Mechanistically, Chidamide reduced m^6^A level of c-Met and its expression by downregulating METTL3 and WTAP [49]. Liu et al. found that METTL3-mediated autophagy is involved in NSCLC resistance to gefitinib, and further mechanistic studies revealed that METTL3 in NSCLC regulates autophagy-related gene expression such as ATG5, ATG7, LC3B, and SQSTM1 to promote cell survival in an m^6^A manner [50]. Similarly, METTL3 overexpression was positively correlated with MET in gefitinib-resistant LUAD. Further studies revealed that METTL3 regulates MET expression, thereby synergistically activating the downstream PI3K/AKT pathway and reducing the sensitivity of LUAD to gefitinib [51]. Conversely, METTL3 knockdown has also been reported to increase NSCLC resistance to Cisplatin [52].

## Effects of METTL3 on the glycolysis of lung cancer

Abnormal glucose metabolism has been reported to facilitate malignant tumor initiation and development, which is one of the key energy metabolism features of tumors [8, 53]. Recent findings have revealed that circPUM1 upregulated the expression levels of glucose transporter 1(GLUT1) and hexokinase-2 (HK2) to promote the glycolysis of lung cancer by upregulating METTL3 [31].

Different from the oxidative phosphorylation of mitochondria in normal cells, tumor cells mainly rely on aerobic glycolysis for energy supply, a phenomenon called "Warburg effect" [54, 55], which provides a beneficial environment for tumor cell growth. The study has shown that METTL3 could activate ABHD11-AS1/EZH2/KLF4 axis to downregulate the expression of transcription factor Kruppel-like factor4 (KLF4), enhancing the Warburg effect [30]. Besides, METTL3 might also be involved in other energy mechanism pathways, such as lipid metabolism, amino acid metabolism, which needs to be further explored.

## Effects of METTL3 on angiogenesis of lung cancer

Angiogenesis, a typical feature of the malignant progression of tumor cells, is a complex biological process by which new capillaries grow from preexisting vessels, providing oxygen and nutrients for the malignant progression of tumors [8, 56, 57]. METTL3 was reported to regulate the expression of let-7e-5p and miR-18a-5p to significantly improve endothelial cells (ECs) biological functions that facilitate neovascularization in limb ischemia and myocardial infarction mouse models [58]. Thus, METTL3 can serve as an important regulator of angiogenesis. Similarly, METTL3 also regulated miR-143-3p/VASH1 axis to enhance the angiogenesis ability of lung cancer cells in an m^6^A manner [59].

## Effects of METTL3 on the tumor microenvironment of lung cancer

The tumor microenvironment (TME) is composed of cancer cells, cancer stem cells, endothelial cells, pericytes, cancer-associated fibroblasts, immune and inflammatory cells, as well as extracellular components such as vascular endothelial-derived growth factor (VEGF), ERGF etc., participating in tumor growth, invasion, metastasis, and drug resistance [8, 27].

**Fig. 2:**
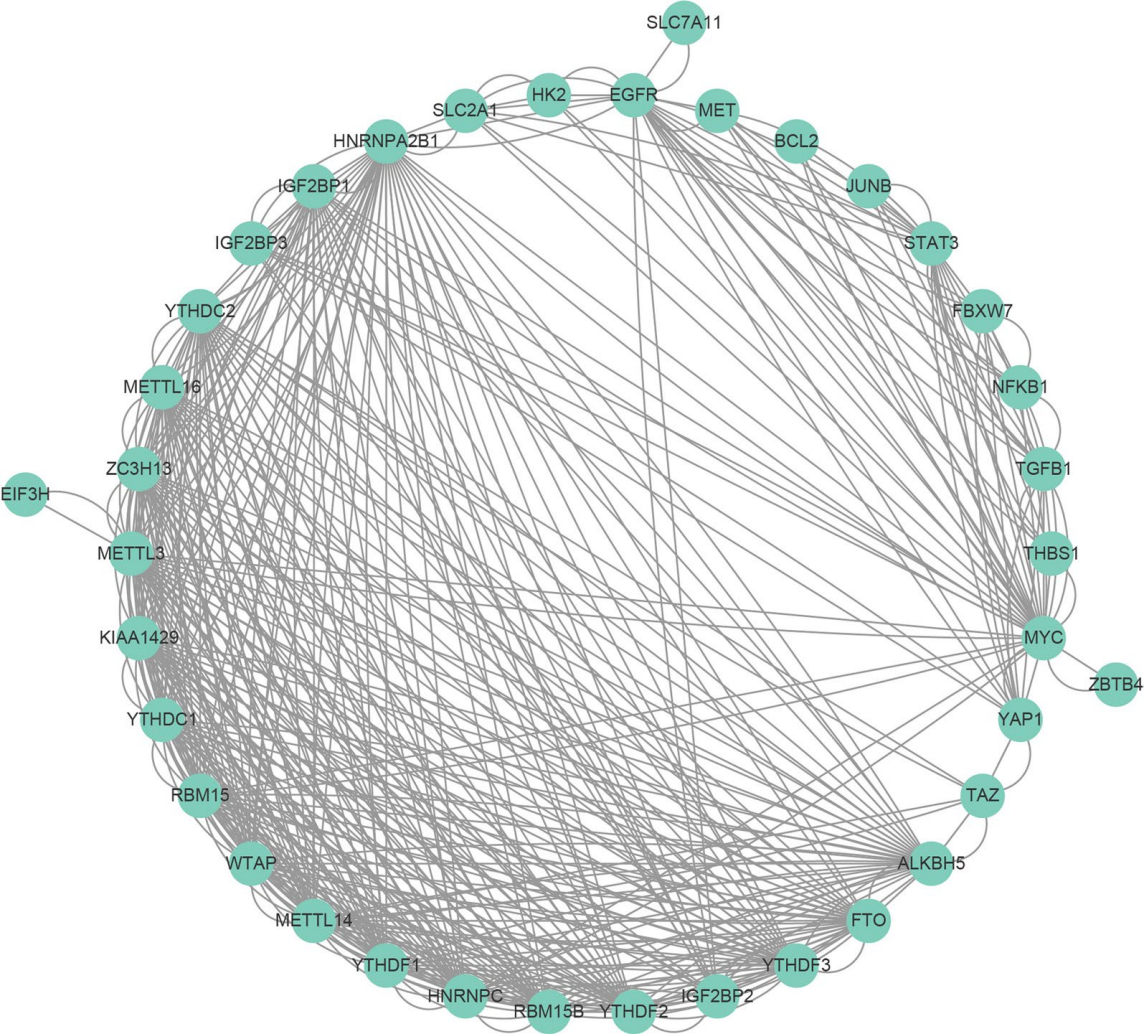
The establishment of the protein–protein interaction network for m^6^A regulators and its associated genes in lung cancer on the basis of String database

METTL3 depletion in macrophages reshaped the TME by increasing M1- and M2-like tumor-associated macrophages (TAMs) and regulatory T (Treg) cell infiltration in vivo, resulting in tumor growth, metastasis, and drug resistance. Mechanistically, ablation of METTL3 in macrophages inhibits the YTHDF1-mediated SPRED2 translation to upregulate ERK expression to activate NF-κB and STAT3 signaling [60]. Thus, METTL3 plays a key role in the TME. However, the molecular mechanisms involved in the TME are quite complex, and METTL3-mediated remodelling of the TME also only reveals the tip of the iceberg where m^6^A methylation participates in the formation of the TME (Fig. 2).

## Effects of METTL3 on the prognosis of lung cancer

More and more studies have demonstrated that METTL3 is strongly linked to the prognosis of lung cancer. However, whether METTL3 can precisely reflect clinical outcomes is controversial. The retrospective study conducted by Liu et al. evaluated the relationship between METTL3 and the prognosis of lung cancer through meta-analysis and bioinformatic analysis [61]. The result has shown that METTL3 overexpression is closely related to the prognosis of various tumors and could serve as a potential tumor biomarker [61].

By analyzing 22 immune cell types, Xu et al. identified that T follicular helper cell signature (risk core) could serve as an independent prognostic factor in patients with lung squamous cell carcinoma (LUSC) [62]. LUSC patients were separated into low-risk and high-risk groups based on this risk core. Interestingly, low-risk groups exhibited a worse OS, in which the expression of METTL3, HNRNPC, ALKBH5, and KIAA1429 was upregulated. While high-risk groups in which m^6^A-related regulators is downregulated better respond to chemotherapies and immunotherapies, suggesting METTL3 overexpression may predict a poor prognosis in LUSC patients [62].

The study by Zhang et al. has shown that METTL3 overexpression in LUAD was strongly related to better OS and progression free survival (PFS) [63]. Hence, METTL3 could serve as the protective gene in LUAD. However, another study suggested the opposite conclusion that METTL3 overexpression was negatively associated with LUAD prognosis [64].

### Construction of a risk score model and lung cancer prognosis

METTL3, together with other m^6^A-related regulators, participates synergistically in an m^6^A methylation. Therefore, it is less reliable to estimate the prognostic value of METTL3 in lung cancer. Hence, based on multiple-gene signatures, the risk score model is constructed to reflect the prognosis of patients with lung cancer more reasonably. Zhu et al. found that nothing in the six m^6^A-related regulators is a prognostic risk factor for lung cancer [65]. The risk score model of six genes was built through bioinformatics analysis, which is significantly associated with clinicopathological features and survival outcomes, serving as an independent predictor of prognosis in LUAD [65]. Similarly, based on eight m^6^A regulators, an optimal prognostic gene risk score model was constructed by Liu et al., which could serve as an independent prognostic factor in LUAD [66]. Additionally, the risk score model constructed by three risk genes (METTL3, YTHDC1, and HNRNPC) also did well in predicting the prognosis of LUSC patients [66]. Moreover, Zhuang et al. constructed the risk score model by using ten m^6^A regulators, which were strongly related to clinicopathological characteristics and could be used to be an independent risk factor in LUAD [67]. Unfortunately, this model was not applicable to LUSC.

Gene alternative splicing (GAS) can be explained by the production of multiple mRNA isoforms from a single gene, which regulates gene expression at the posttranscriptional level and plays a crucial role in the development of diverse diseases, including cancer [68–71]. The risk signature of the m^6^A-associated GAS events was constructed by Zhao et al., which could serve as an independent prognostic risk factor in LUAD and LUSC [72]. Of note, METTL3, HNRNPC, and RBM15, as the splicing factors, can also be directly involved in GAS events in NSCLC [72].

## Relationship between METTL3 and noncoding RNA in lung cancer

Noncoding RNA is functional RNA that is not translated into proteins but can regulate gene expression [73, 74]. According to length, noncoding RNA can be divided into short-chain noncoding RNA (siRNA, miRNA, piRNA) and long-chain noncoding RNA (LncRNA) [75, 76].

In mammals, miRNA can regulate the expression of target genes at the posttranscriptional level through incomplete complementary pairing with mRNA 3'UTR. Interestingly, 3'UTR is also where m^6^A modification is enriched [75]. Studies have shown that approximately 67% of transcripts in the 3'UTR with m^6^A modification contain at least one miRNA binding site [18]. Hence, METTL3-mediated m^6^A methylation modification has a high link with miRNA. Functionally, both can regulate critical oncogene expression and influence tumor progression. Additionally, it has been reported that nine m^6^A-mediated miRNA were identified in human bronchial epithelial cells (HBEs) treated with arsenite by using the Venn diagram and KEGG analysis [77], which might regulate crucial pathways related to cell proliferation and apoptosis, including the P53 pathway, mTOR pathway, and MAPK pathway, suggesting that miRNA could serve as the pivotal bridge by which METTL3-mediated m^6^A facilitates to cell proliferation or apoptosis in HBEs treated with arsenite. Table 2 lists the relationships between METTL3 and noncoding RNA in lung cancer.

**Table 2:** The relationships between METTL3 and noncoding RNA

Up-regulator	Target	Mechanism	Biological function	Refs
miR-4443	METTL3	Enhance FSP1 expression	Promote drug resistance	[52]
circPUM1	miR-590-5p	Upregulate the expression of METTL3	Promote cell growth and glycolysis	[31]
METTL3	Lnc RNA MALAT1	Activate MALAT1-miR-1914-3p-YAP axis	Promote drug resistance and metastasis	[38]
METTL3	Lnc RNA ABHD11-AS1	Enhance the stability of ABHD11‐AS1 transcript	Promote the proliferation and Warburg effect of NSCLC cells	[30]
METTL3	miR-143-3p	Increase the splicing of precursor miR-143-3p	Promote angiogenesis and brain metastasis of lung cancer	[59]
METTL3	miR-1246	Activate miR-1246/PEG3 axis	Promote NSCLC progression	[104]
miR-338-5p	METTL3	Decrease C-myc expression	Inhibit lung cancer malignant progression	[105]
miR-33a	METTL3	Inhibit the expression of METTL3	Suppress NSCLC cell proliferation	[32]
miR-600	METTL3	Downregulate the expression of METTL3	Inhibit lung cancer progression	[102]

In addition, the METTL3-YTHDC1 participates in the back-splicing of some circRNAs and affects their biogenesis. For example, YTHDF3/eIF4G2 regulates its translation by recognizing a specific m^6^A site on circ-ZNF609 [78]. It has been reported that circPUM1 knockdown inhibits NSCLC cell growth and glycolysis in vivo and vitro. Further study has shown that circPUM1 sponges miR-590-5p, which can directly target METTL3 and downregulate its expression. Liu et al. identified a novel circIGF2BP3 that is overexpressed in NSCLC and inhibits CD8^+^ T cell infiltration [79]. Mechanistically, METTL3 promotes PKP3 mRNA to form a protein-RNA complex with FXR1 to stabilize OTUB1 mRNA by regulating the circIGF2BP3/PKP3 axis. OTUB1 upregulates its expression by inhibiting PD-L1 ubiquitination in NSCLC cells to induce immune escape and resistance to PD-1 inhibitors.

## Recent advances in targeting METTL3

Accumulating evidence has shown that METTL3 plays a crucial role in the tumorigenesis of lung cancer, dependent or independent of m^6^A modification. It could act as a potential therapeutic target (Table 3).

**Table 3:** METTL3 activators and inhibitors

Drug	Activator/Inhibitor	Target	Mechanism	Biological function	Refs
IL-37	Activator	METTL3, YTHDC3, METTL14, WTAP, ALKBH5, Wnt5a/5b pathway	Upregulate METTL3, YTHDC3 and downregulate METTL14, WTAP, ALKBH5	Inhibit tumor growth	[94]
Compound 1/2/3/4	Activator	METTL3	Lower the energy barrier of the substrate RNAs methylation reaction	Increase the total m^6^A level	[95]
ATTM	Activator	METTL3, FTO	Increase PRPF6 m^6^A methylation level	Promote cell growth, proliferation and invasion	[103]
STM2457	Inhibitor	METTL3	Decrease the level of leukaemogenic mRNAs m^6^A methylation	Inhibit tumor growth and eliminate stem cell subpopulations of AML	[107]
Simvastatin	Inhibitor	METTL3	Downregulate METTL3 expression and regulate the m^6^A level of EZH2 mRNA	Inhibit the EMT process of lung cancer	[83]
Chidamide	Inhibitor	METTL3, WTAP	Downregulate METTL3 and WTAP expression	Decrease c-Met RNA m^6^A methylation level and inhibit NSCLC drug resistance	[91]
Compound 2/ 7	Inhibitor	SAM	Serve as SAM-competitive inhibitor of METTL3	Decrease the total m^6^A level	[82]

### METTL3 inhibitors

As the sole catalytic subunit in the methyltransferase complex, METTL3 is involved in different aspects of tumor progression, such as cell proliferation, invasion, migration, metastasis, tumor environment, cancer stem cells, and drug resistance [43, 80, 81].

Because adenosine could serve as a SAM-competitive inhibitor of METTL3, Bedi et al. identified seven compounds from among 4000 adenosine analogs and derivatives using high-throughput docking into METTL3, two of which (compounds 2 and 7) showed good ligand efficiency [82]. Additionally, simvastatin has been reported to exert anti-tumor activity in various cancers, including lung cancer. The study by Chen et al. has indicated that simvastatin suppresses cell proliferation, migration, invasion, metastasis and EMT by reducing EZH2 expression via downregulating METTL3 in lung cancer [83].

### Drug combination

In order to overcome multiple drug resistance and prolong patient survival, drug combination is becoming the mainstream of lung cancer therapy [84, 85]. Recent evidence has shown that two different targeted agents for lung cancer could block numerous targets on the signal transduction pathway to prevent the malignant progression of lung cancer, thereby achieving better clinical efficacy [86–88]. Chidamide is a novel small molecular inhibitor targeting HDAC1/2/3/10 [89]. Interestingly, histone deacetylase inhibitors (HDACIs) combined with other agents strongly improved anti-tumor activities [90–93]. Recently, Ding et al. revealed that Chidamide could make NSCLC cells more sensitive to Crizotinib in vivo. Mechanistically, Chidamide can decrease the stability and translational efficiency of METTL3 and WTAP transcripts to downregulate the m^6^A level and expression of c-Met [49].

### Others

METTL3 overexpression is common in NSCLC and dramatically accelerates the transcriptional efficiency of key oncogenes, resulting in NSCLC malignancy [42, 43]. However, it has also been shown that METTL3 overexpression is associated with a better prognosis in NSCLC patients. Based on bioinformatics analysis, Liu et al. constructed a risk score model with eight m^6^A methylation regulators, including METTL3, which could better respond to the clinical outcomes of LUAD and LUSC patients. METTL3 acted as a protective gene in this model and was commonly enriched in the low-risk group [66]. Similarly, Zhang et al. found that METTL3 overexpression was positively correlated with OS in LUAD samples from The Cancer Genome Atlas (TCGA) [63]. Zhu et al. constructed a risk score model based on six m^6^A methylation regulators that could better predict the clinicopathological characteristics of LUAD patients, and METTL3 acted as a tumor suppressor in this model [65].

It has been reported that Interleukin 37 (IL-37) increases METTL3 expression to upregulate the total m^6^A level, inhibiting A549 cell proliferation. Furthermore, IL-37 has anti-tumor activity by targeting the Wnt5a/5b pathway [94]. Therefore, Selberg et al. performed a virtual screening based on the crystal structure of the methyltransferase complex. It turned out that four small molecule compounds enhance its methyltransferase activity by specifically binding METTL3, upregulating the total m^6^A level in RNA in cells [95]. Mechanically, the compound increases SAM affinity for METTL3 and lowers the energy barrier of the m^6^A methylation reaction by interaction with SAM in the active center of METTL3 [95].

## METTL3-METTL14-WTAP complex in lung cancer

**Fig. 3:**
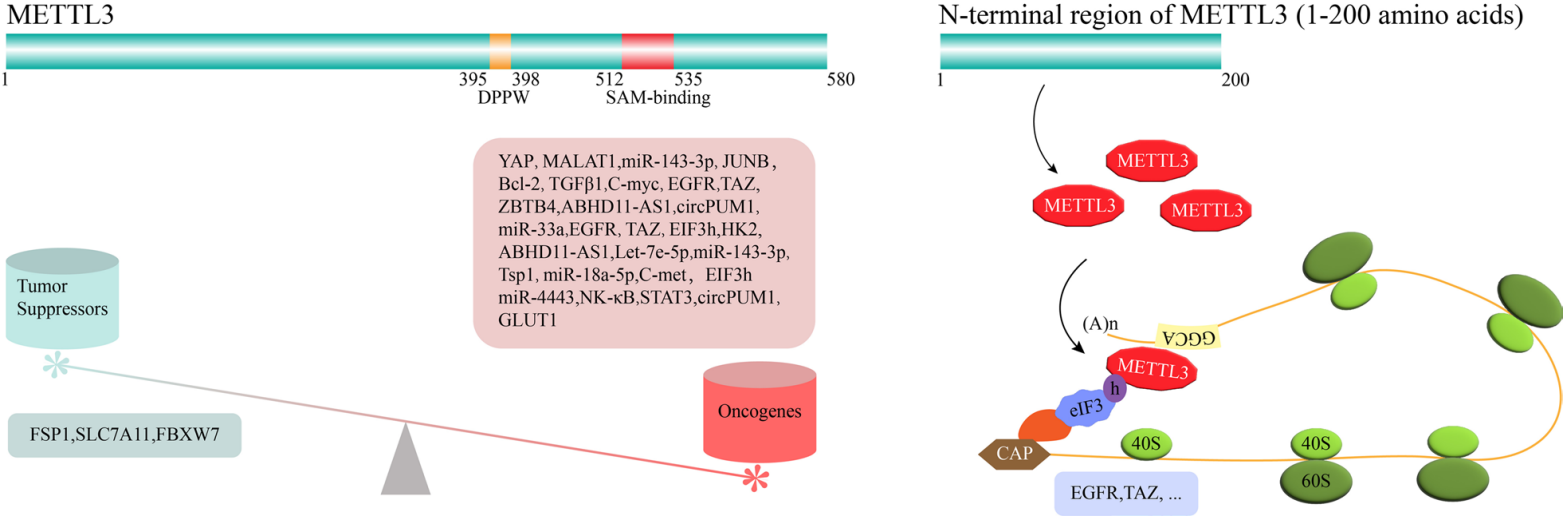
METTL3 regulates tumor suppressors and oncogenes expression to promote lung cancer progression in an m6A manner (left). In addition, METTL3 also accelerate the translation efficiency of key oncogenes to affect lung cancer progression independent of its methyltransferase activity (right)

METTL3 is the first identified methyltransferase and has sole enzymatic activity. However, METTL3 interacts with METTL14 to form a heterodimer with the highest enzymatic activity and a better preference for substrate RNAs [44]. However, WTAP binds to the METTL3-METTL14 heterodimer to form a METTL3-METTL14-WTAP complex localized at the nuclear speckle, thereby affecting the total m^6^A level [96]. Interestingly, METTL3, as the only catalytically active subunit of the methyltransferase complex, promotes the development of lung cancer dependent or independent of catalytic activity. On the one hand, METTL3 affects m^6^A levels of oncogenes or tumor suppressors, thereby regulating their expression to promote tumor progression [39, 81]. On the other hand, METTL3 interacts with eIF3h to form RNA looping independent on catalytic activity, which greatly accelerates the translation efficiency of polyribosomes and upregulates the expression of key oncogenes, such as EGFR and TAZ [42, 43] (Fig. 3).

It has been reported that METTL14 depletion significantly downregulates m^6^A levels in tumor stromal cells, thereby decreasing CD8^+^ T cell infiltration and increasing dysfunctional T cells, leading to tumor growth [79]. Further study revealed that the METTL14-YTHDF2 axis maintained the balance between cytotoxic CD8^+^ T cells and dysfunctional T cells, and METTL14-depleted TAMs overexpressing Epstein-Barr virus-induced protein 3 (Ebi3) transcripts inhibited the anti-tumor activity of CD8^+^ T cells, which resulted in the conversion of CD8^+^ T cells to dysfunctional T cells. Furthermore, METTL14 is overexpressed in NSCLC cell lines and induces the EMT process [97]. Mechanistically, METTL14 knockdown downregulates Twist expression to inhibit the AKT pathway and upregulate E-cadherin, thereby inhibiting NSCLC cell migration. Of note, Li et al., based on TCGA and GEO databases, found that METTL14 was downregulated in lung cancer tissues compared to normal tissues. And METTL14 overexpression inhibited lung cancer growth and metastasis in vivo and in vitro through the miR-30c-1-3p/MARCKSL1 axis [98].

In 2000, Little et al. first identified WTAP using a yeast two-hybrid assay [99]. WTAP, a widespread nuclear protein, can specifically bind to the WT1 protein to co-localize at the nuclear speckle [99]. In addition, WTAP can also act as a splicing factor and participate in alternative splicing of specific a subset of m^6^A-modified RNAs. It has been reported that LncRNA PCGEM1 is highly expressed in NSCLC and promotes cell growth [100]. Mechanistically, LncRNA PCGEM1 sponges miR-433-3p to upregulate WTAP expression and accelerate NSCLC progression. Intriguingly, METTL3 levels are critical for WTAP homeostasis, and METTL3 knockdown or overexpression both upregulate WTAP expression [101]. Mechanistically, METTL3 overexpression increases WTAP mRNA translation and protein stability independent of its catalytic activity. In contrast, METTL3 knockdown increases WTAP mRNA levels and eventually upregulates the expression of WTAP. Notably, WTAP overexpression is insufficient to promote tumor progression when functional METTL3 is absent, implying that WTAP must depend on the METTL3-METTL14 complex to exert oncogenic activity [101].

## Conclusions

Accumulating studies have shown that METTL3 plays a crucial role in the occurrence and development of lung cancer. METTL3 participates in cell proliferation, invasion, migration, metastasis, angiogenesis, glycolysis, drug resistance, and tumor microenvironment, dependent or not on catalytic activity. Thus, METTL3 could act as a potential therapeutic target for lung cancer. Notably, m^6^A regulators include methyltransferases and demethylases, while METTL3, as the sole methyltransferase with enzymatic activity, does not ultimately determine the global m^6^A level, suggesting that m6A levels may be regulated by specific patterns that require further investigation.

On the one hand, METTL3-mediated m^6^A methylation regulates the expression of oncogenes or tumor suppressors, influencing the malignant progression of lung cancer. On the other hand, METTL3 interacts with eIF3h to form RNA looping, significantly increasing the translation efficiency of key oncogenes. The combined effect of the two may determine the development of lung cancer. Intriguingly, either METTL3 knockdown or overexpression can regulate WTAP expression, suggesting that they can interact with each other, and act as upstream regulators of each other. Therefore, the regulatory network with METTL3 as the core in lung cancer is extremely complex and involves many molecular mechanisms.

A large number of studies have shown that METTL3 interacts with noncoding RNA to regulate the expression of downstream genes, thereby affecting the progression of lung cancer. Among noncoding RNA, especially circRNAs, the METTL3-YTHDC1 participates in circRNAs biogenesis and regulates the translation of specific m^6^A-modified circRNAs.

Notably, there is solid evidence that METTL3 can act as an oncogene in lung cancer cell lines but as a tumor suppressor in tumor stromal cells. For example, METTL3 deletion in macrophages can promote tumor growth, metastasis, and drug resistance by increasing M1- and M2-TAM and Treg infiltration in tumors and reshaping the tumor microenvironment. In addition, METTL14-deficient macrophages significantly downregulated the global m^6^A level to reduce CD8^+^ T cells and increase dysfunctional T cells, promoting tumor growth. In tumor stromal cells, especially TAM, METTL3 or METTL14 can serve as a protective genes for lung cancer. To our knowledge, this is the first time to propose that METTL3 and METTL14 play opposite roles in lung cancer cells and tumor stromal cells, precisely explaining why high levels of m^6^A  methylation  can be predictive of better prognosis in NSCLC patients.

## Data Availability

Not applicable.

## Abbreviations

ALKBH5: Alkb homologue 5
ATTM: Ammonium tetrathiomolybdate
BRD4: Bromodomain-containing protein
CSCs: Cancer stem cells
EGFR: Epidermal growth factor receptor
EMT: Epithelial-mesenchymal transition
ECs: Endothelial cells
FTO: Fat mass and obesity-associated protein
GEO: Gene Expression Omnibus
GAS: Gene alternative splicing
GLUT1: Glucose transporter 1
KLF4: Kruppel-like factor4
HBEs: Human bronchial epithelial cells
HGF: Hepatocyte growth factor
HBXIP: Hepatitis B X-interacting protein
HK2: Hexokinase-2
HDGF: Hepatoma-derived growth factor
IL-37: Interleukin 37
LUAD: Lung adenocarcinoma
LUSC: Lung squamous cell carcinoma
Lnc RNA: Long noncoding RNA
METTL3: Methyltransferase-like 3
NSCLC: Non-small cell lung cancer
OS: Overall survival
RFS: Progression free survival
LASSO: Least absolute shrinkage and selection operator
PM_2.5_: Particulate matter 2.5
SCLC: Small cell lung cancer
SNAI1: Snail family transcriptional repressor 1
SAM: *S*-Adenosylmethionine
SOCS2: Suppressor of cytokine signaling 2
TCGA: The Cancer Genome Atlas
TME: Tumor environment
TAZ: Transcriptional coactivator with PDZ-binding motif
VEGF: Vascular endothelial-derived growth factor
YAP: Yes-associated protein
3'UTR: 3' Untranslated region

## References

[1] Sheikh M, Mukeriya A, Shangina O, Brennan P, Zaridze D (2021). Postdiagnosis smoking cessation and reduced risk for lung cancer progression and mortality: a prospective cohort study. Ann Intern Med.

[2] Siegel RL, Miller KD, Fuchs HE, Jemal A (2021). Cancer statistics, 2021. CA Cancer J Clin.

[3] Thai AA, Solomon BJ, Sequist LV, Gainor JF, Heist RS (2021). Lung cancer. Lancet.

[4] Zhang Y, Wang DC, Shi L, Zhu B, Min Z, Jin J (2017). Genome analyses identify the genetic modification of lung cancer subtypes. Semin Cancer Biol.

[5] Morgan E, Arnold M, Rutherford MJ, Bardot A, Ferlay J, De P, Engholm G, Jackson C, Little A, Saint-Jacques N (2021). The impact of reclassifying cancers of unspecified histology on international differences in survival for small cell and non-small cell lung cancer (ICBP SurvMark-2 project). Int J Cancer.

[6] Oudkerk M, Liu S, Heuvelmans MA, Walter JE, Field JK (2021). Lung cancer LDCT screening and mortality reduction—evidence, pitfalls and future perspectives. Nat Rev Clin Oncol.

[7] Leon G, MacDonagh L, Finn SP, Cuffe S, Barr MP (2016). Cancer stem cells in drug resistant lung cancer: targeting cell surface markers and signaling pathways. Pharmacol Ther.

[8] Hanahan D, Weinberg RA (2011). Hallmarks of cancer: the next generation. Cell.

[9] Wang T, Kong S, Tao M, Ju S (2020). The potential role of RNA N6-methyladenosine in cancer progression. Mol Cancer.

[10] Hogg SJ, Beavis PA, Dawson MA, Johnstone RW (2020). Targeting the epigenetic regulation of antitumour immunity. Nat Rev Drug Discov.

[11] Wątroba M, Dudek I, Skoda M, Stangret A, Rzodkiewicz P, Szukiewicz D (2017). Sirtuins, epigenetics and longevity. Ageing Res Rev.

[12] Skvortsova K, Iovino N, Bogdanović O (2018). Functions and mechanisms of epigenetic inheritance in animals. Nat Rev Mol Cell Biol.

[13] Zeng C, Huang W, Li Y, Weng H (2020). Roles of METTL3 in cancer: mechanisms and therapeutic targeting. J Hematol Oncol.

[14] Li J, Yang X, Qi Z, Sang Y, Liu Y, Xu B, Liu W, Xu Z, Deng Y (2019). The role of mRNA m(6)A methylation in the nervous system. Cell Biosci.

[15] Xu J, Liu Y, Liu J, Xu T, Cheng G, Shou Y, Tong J, Liu L, Zhou L, Xiao W (2020). The identification of critical m(6)A RNA methylation regulators as malignant prognosis factors in prostate adenocarcinoma. Front Genet.

[16] Desrosiers R, Friderici K, Rottman F (1974). Identification of methylated nucleosides in messenger RNA from Novikoff hepatoma cells. Proc Natl Acad Sci USA.

[17] Wei CM, Gershowitz A, Moss B (1975). Methylated nucleotides block 5' terminus of HeLa cell messenger RNA. Cell.

[18] Meyer KD, Saletore Y, Zumbo P, Elemento O, Mason CE, Jaffrey SR (2012). Comprehensive analysis of mRNA methylation reveals enrichment in 3' UTRs and near stop codons. Cell.

[19] Ke S, Alemu EA, Mertens C, Gantman EC, Fak JJ, Mele A, Haripal B, Zucker-Scharff I, Moore MJ, Park CY (2015). A majority of m6A residues are in the last exons, allowing the potential for 3' UTR regulation. Genes Dev.

[20] Jia G, Fu Y, Zhao X, Dai Q, Zheng G, Yang Y, Yi C, Lindahl T, Pan T, Yang Y-G (2011). N6-methyladenosine in nuclear RNA is a major substrate of the obesity-associated FTO. Nat Chem Biol.

[21] Zheng G, Dahl JA, Niu Y, Fedorcsak P, Huang C-M, Li CJ, Vågbø CB, Shi Y, Wang W-L, Song S-H (2013). ALKBH5 is a mammalian RNA demethylase that impacts RNA metabolism and mouse fertility. Mol Cell.

[22] Chen X-Y, Zhang J, Zhu J-S (2019). The role of m6A RNA methylation in human cancer. Mol Cancer.

[23] Meyer KD, Jaffrey SR (2017). Rethinking m(6)A readers, writers, and erasers. Annu Rev Cell Dev Biol.

[24] Lan Q, Liu PY, Haase J, Bell JL, Hüttelmaier S, Liu T (2019). The critical role of RNA m(6)A methylation in cancer. Cancer Res.

[25] Huang H, Weng H, Chen J (2020). m(6)A modification in coding and non-coding RNAs: roles and therapeutic implications in cancer. Cancer Cell.

[26] Wang Q, Geng W, Guo H, Wang Z, Xu K, Chen C, Wang S (2020). Emerging role of RNA methyltransferase METTL3 in gastrointestinal cancer. J Hematol Oncol.

[27] Hanahan D (2022). Hallmarks of cancer: new dimensions. Cancer Discov.

[28] Siegel RL, Miller KD, Fuchs HE, Jemal A (2022). Cancer statistics, 2022. CA Cancer J Clin.

[29] Liu Z, Wang T, She Y, Wu K, Gu S, Li L, Dong C, Chen C, Zhou Y (2021). N(6)-methyladenosine-modified circIGF2BP3 inhibits CD8(+) T-cell responses to facilitate tumor immune evasion by promoting the deubiquitination of PD-L1 in non-small cell lung cancer. Mol Cancer.

[30] Xue L, Li J, Lin Y, Liu D, Yang Q, Jian J, Peng J (2021). m(6) A transferase METTL3-induced lncRNA ABHD11-AS1 promotes the Warburg effect of non-small-cell lung cancer. J Cell Physiol.

[31] Li M, Wang Q, Zhang X, Yan N, Li X (2021). CircPUM1 promotes cell growth and glycolysis in NSCLC via up-regulating METTL3 expression through miR-590–5p. Cell Cycle.

[32] Du M, Zhang Y, Mao Y, Mou J, Zhao J, Xue Q, Wang D, Huang J, Gao S, Gao Y (2017). MiR-33a suppresses proliferation of NSCLC cells via targeting METTL3 mRNA. Biochem Biophys Res Commun.

[33] Wu Y, Chang N, Zhang Y, Zhang X, Xu L, Che Y, Qiao T, Wu B, Zhou Y, Jiang J (2021). METTL3-mediated m(6)A mRNA modification of FBXW7 suppresses lung adenocarcinoma. J Exp Clin Cancer Res.

[34] Ma L, Chen T, Zhang X, Miao Y, Tian X, Yu K, Xu X, Niu Y, Guo S, Zhang C (2021). The m(6)A reader YTHDC2 inhibits lung adenocarcinoma tumorigenesis by suppressing SLC7A11-dependent antioxidant function. Redox Biol.

[35] Wanna-Udom S, Terashima M, Lyu H, Ishimura A, Takino T, Sakari M, Tsukahara T, Suzuki T (2020). The m6A methyltransferase METTL3 contributes to Transforming Growth Factor-beta-induced epithelial-mesenchymal transition of lung cancer cells through the regulation of JUNB. Biochem Biophys Res Commun.

[36] Deng X, Su R, Feng X, Wei M, Chen J (2018). Role of N(6)-methyladenosine modification in cancer. Curr Opin Genet Dev.

[37] Teng PC, Liang Y, Yarmishyn AA, Hsiao YJ, Lin TY, Lin TW, Teng YC, Yang YP, Wang ML, Chien CS (2021). RNA modifications and epigenetics in modulation of lung cancer and pulmonary diseases. Int J Mol Sci.

[38] Jin D, Guo J, Wu Y, Du J, Yang L, Wang X, Di W, Hu B, An J, Kong L (2019). m(6)A mRNA methylation initiated by METTL3 directly promotes YAP translation and increases YAP activity by regulating the MALAT1-miR-1914-3p-YAP axis to induce NSCLC drug resistance and metastasis. J Hematol Oncol.

[39] Li J, Xie G, Tian Y, Li W, Wu Y, Chen F, Lin Y, Lin X, Wing-Ngor AuS, Cao J (2022). RNA m(6)A methylation regulates the dissemination of cancer cells via modulating expression and membrane localization of β-catenin. Mol Ther.

[40] Cayir A, Barrow TM, Guo L, Byun H-M (2019). Exposure to environmental toxicants reduces global N6-methyladenosine RNA methylation and alters expression of RNA methylation modulator genes. Environ Res.

[41] Cheng C, Wu Y, Xiao T, Xue J, Sun J, Xia H, Ma H, Lu L, Li J, Shi A (2021). METTL3-mediated m(6)A modification of ZBTB4 mRNA is involved in the smoking-induced EMT in cancer of the lung. Mol Ther Nucleic Acids.

[42] Lin S, Choe J, Du P, Triboulet R, Gregory RI (2016). The m(6)A methyltransferase METTL3 promotes translation in human cancer cells. Mol Cell.

[43] Choe J, Lin S, Zhang W, Liu Q, Wang L, Ramirez-Moya J, Du P, Kim W, Tang S, Sliz P (2018). mRNA circularization by METTL3-eIF3h enhances translation and promotes oncogenesis. Nature.

[44] Liu J, Yue Y, Han D, Wang X, Fu Y, Zhang L, Jia G, Yu M, Lu Z, Deng X (2014). A METTL3-METTL14 complex mediates mammalian nuclear RNA N6-adenosine methylation. Nat Chem Biol.

[45] Szakács G, Paterson JK, Ludwig JA, Booth-Genthe C, Gottesman MM (2006). Targeting multidrug resistance in cancer. Nat Rev Drug Discov.

[46] Vasan N, Baselga J, Hyman DM (2019). A view on drug resistance in cancer. Nature.

[47] Westover D, Zugazagoitia J, Cho BC, Lovly CM, Paz-Ares L (2018). Mechanisms of acquired resistance to first- and second-generation EGFR tyrosine kinase inhibitors. Ann Oncol.

[48] Engelman JA, Zejnullahu K, Mitsudomi T, Song Y, Hyland C, Park JO, Lindeman N, Gale C-M, Zhao X, Christensen J (2007). MET amplification leads to gefitinib resistance in lung cancer by activating ERBB3 signaling. Science.

[49] Ding N, You A, Tian W, Gu L, Deng D (2020). Chidamide increases the sensitivity of non-small cell lung cancer to crizotinib by decreasing c-MET mRNA methylation. Int J Biol Sci.

[50] Liu S, Li Q, Li G, Zhang Q, Zhuo L, Han X, Zhang M, Chen X, Pan T, Yan L (2020). The mechanism of m(6)A methyltransferase METTL3-mediated autophagy in reversing gefitinib resistance in NSCLC cells by β-elemene. Cell Death Dis.

[51] Gao F, Wang Q, Zhang C, Zhang C, Qu T, Zhang J, Wei J, Guo R (2021). RNA methyltransferase METTL3 induces intrinsic resistance to gefitinib by combining with MET to regulate PI3K/AKT pathway in lung adenocarcinoma. J Cell Mol Med.

[52] Song Z, Jia G, Ma P, Cang S (2021). Exosomal miR-4443 promotes cisplatin resistance in non-small cell lung carcinoma by regulating FSP1 m6A modification-mediated ferroptosis. Life Sci.

[53] Ganapathy-Kanniappan S, Geschwind J-FH (2013). Tumor glycolysis as a target for cancer therapy: progress and prospects. Mol Cancer.

[54] Icard P, Shulman S, Farhat D, Steyaert JM, Alifano M, Lincet H (2018). How the Warburg effect supports aggressiveness and drug resistance of cancer cells?. Drug Resist Updat.

[55] Zhang D, Tang Z, Huang H, Zhou G, Cui C, Weng Y, Liu W, Kim S, Lee S, Perez-Neut M (2019). Metabolic regulation of gene expression by histone lactylation. Nature.

[56] Viallard C, Larrivée B (2017). Tumor angiogenesis and vascular normalization: alternative therapeutic targets. Angiogenesis.

[57] Yetkin-Arik B, Kastelein AW, Klaassen I, Jansen CHJR, Latul YP, Vittori M, Biri A, Kahraman K, Griffioen AW, Amant F (2021). Angiogenesis in gynecological cancers and the options for anti-angiogenesis therapy. Biochim Biophys Acta Rev Cancer.

[58] Chamorro-Jorganes A, Sweaad WK, Katare R, Besnier M, Anwar M, Beazley-Long N, Sala-Newby G, Ruiz-Polo I, Chandrasekera D, Ritchie AA (2021). METTL3 regulates angiogenesis by modulating let-7e-5p and miRNA-18a-5p expression in endothelial cells. Arterioscler Thromb Vasc Biol.

[59] Wang H, Deng Q, Lv Z, Ling Y, Hou X, Chen Z, Dinglin X, Ma S, Li D, Wu Y (2019). N6-methyladenosine induced miR-143-3p promotes the brain metastasis of lung cancer via regulation of VASH1. Mol Cancer.

[60] Yin H, Zhang X, Yang P, Zhang X, Peng Y, Li D, Yu Y, Wu Y, Wang Y, Zhang J (2021). RNA m6A methylation orchestrates cancer growth and metastasis via macrophage reprogramming. Nat Commun.

[61] Liu K, Gao Y, Gan K, Wu Y, Xu B, Zhang L, Chen M (2021). Prognostic roles of N6-methyladenosine METTL3 in different cancers: a system review and meta-analysis. Cancer Control.

[62] Xu F, Zhang H, Chen J, Lin L, Chen Y (2020). Immune signature of T follicular helper cells predicts clinical prognostic and therapeutic impact in lung squamous cell carcinoma. Int Immunopharmacol.

[63] Zhang Y, Liu X, Liu L, Li J, Hu Q, Sun R (2020). Expression and prognostic significance of m6A-related genes in lung adenocarcinoma. Med Sci Monit.

[64] Wang H, Zhao X, Lu Z (2021). m(6)A RNA methylation regulators act as potential prognostic biomarkers in lung adenocarcinoma. Front Genet.

[65] Zhu J, Wang M, Hu D (2020). Deciphering N(6)-methyladenosine-related genes signature to predict survival in lung adenocarcinoma. Biomed Res Int.

[66] Liu Y, Guo X, Zhao M, Ao H, Leng X, Liu M, Wu C, Ma J, Zhu J (2020). Contributions and prognostic values of m6 A RNA methylation regulators in non-small-cell lung cancer. J Cell Physiol.

[67] Zhuang Z, Chen L, Mao Y, Zheng Q, Li H, Huang Y, Hu Z, Jin Y (2020). Diagnostic, progressive and prognostic performance of m(6)A methylation RNA regulators in lung adenocarcinoma. Int J Biol Sci.

[68] Coomer AO, Black F, Greystoke A, Munkley J, Elliott DJ (2019). Alternative splicing in lung cancer. Biochim Biophys Acta Gene Regul Mech.

[69] Urbanski LM, Leclair N, Anczuków O (2018). Alternative-splicing defects in cancer: splicing regulators and their downstream targets, guiding the way to novel cancer therapeutics. Wiley Interdiscip Rev RNA..

[70] Wu L, Wu D, Ning J, Liu W, Zhang D (2019). Changes of N6-methyladenosine modulators promote breast cancer progression. BMC Cancer.

[71] Baralle FE, Giudice J (2017). Alternative splicing as a regulator of development and tissue identity. Nat Rev Mol Cell Biol.

[72] Qin F, Cai B, Zhao J, Zhang L, Zheng Y, Liu B, Gao C (2021). Methyltransferase-like protein 14 attenuates mitochondrial antiviral signaling protein expression to negatively regulate antiviral immunity via N(6) -methyladenosine modification. Adv Sci.

[73] Yang F, Yi F, Zheng Z, Ling Z, Ding J, Guo J, Mao W, Wang X, Wang X, Ding X (2012). Characterization of a carcinogenesis-associated long non-coding RNA. RNA Biol.

[74] St Laurent G, Wahlestedt C, Kapranov P (2015). The landscape of long noncoding RNA classification. Trends Genet.

[75] Chen Y, Lin Y, Shu Y, He J, Gao W (2020). Interaction between N(6)-methyladenosine (m(6)A) modification and noncoding RNAs in cancer. Mol Cancer.

[76] Yi YC, Chen XY, Zhang J, Zhu JS (2020). Novel insights into the interplay between m(6)A modification and noncoding RNAs in cancer. Mol Cancer.

[77] Gu S, Sun D, Dai H, Zhang Z (2018). N(6)-methyladenosine mediates the cellular proliferation and apoptosis via microRNAs in arsenite-transformed cells. Toxicol Lett.

[78] Yang Y, Fan X, Mao M, Song X, Wu P, Zhang Y, Jin Y, Yang Y, Chen L-L, Wang Y (2017). Extensive translation of circular RNAs driven by N6-methyladenosine. Cell Res.

[79] Dong L, Chen C, Zhang Y, Guo P, Wang Z, Li J, Liu Y, Liu J, Chang R, Li Y (2021). The loss of RNA N(6)-adenosine methyltransferase Mettl14 in tumor-associated macrophages promotes CD8(+) T cell dysfunction and tumor growth. Cancer Cell.

[80] Lin X, Chai G, Wu Y, Li J, Chen F, Liu J, Luo G, Tauler J, Du J, Lin S (2019). RNA m6A methylation regulates the epithelial mesenchymal transition of cancer cells and translation of Snail. Nat Commun.

[81] Zhang Y, Liu S, Zhao T, Dang C (2021). METTL3-mediated m6A modification of Bcl-2 mRNA promotes non-small cell lung cancer progression. Oncol Rep.

[82] Bedi RK, Huang D, Eberle SA, Wiedmer L, Śledź P, Caflisch A (2020). Small-molecule inhibitors of METTL3, the major human epitranscriptomic writer. ChemMedChem.

[83] Chen W-W, Qi J-W, Hang Y, Wu J-X, Zhou X-X, Chen J-Z, Wang J, Wang H-H (2020). Simvastatin is beneficial to lung cancer progression by inducing METTL3-induced m6A modification on EZH2 mRNA. Eur Rev Med Pharmacol Sci.

[84] Li W, Zhang H, Assaraf YG, Zhao K, Xu X, Xie J, Yang DH, Chen ZS (2016). Overcoming ABC transporter-mediated multidrug resistance: molecular mechanisms and novel therapeutic drug strategies. Drug Resist Updat.

[85] Li F, Wang H, Huang H, Zhang L, Wang D, Wan Y (2020). m6A RNA methylation regulators participate in the malignant progression and have clinical prognostic value in lung adenocarcinoma. Front Genet.

[86] Losanno T, Rossi A, Maione P, Napolitano A, Gridelli C (2016). Anti-EGFR and antiangiogenic monoclonal antibodies in metastatic non-small-cell lung cancer. Expert Opin Biol Ther.

[87] Tang H, Liu Y, Wang C, Zheng H, Chen Y, Liu W, Chen X, Zhang J, Chen H, Yang Y (2019). Inhibition of COX-2 and EGFR by melafolone improves anti-PD-1 therapy through vascular normalization and PD-L1 downregulation in lung cancer. J Pharmacol Exp Ther.

[88] Zhang Z, Zhang Y, Luo F, Ma Y, Fang W, Zhan J, Li S, Yang Y, Zhao Y, Hong S (2020). Dual blockade of EGFR and VEGFR pathways: results from a pilot study evaluating apatinib plus gefitinib as a first-line treatment for advanced EGFR-mutant non-small cell lung cancer. Clin Transl Med.

[89] Qiu T, Zhou L, Zhu W, Wang T, Wang J, Shu Y, Liu P (2013). Effects of treatment with histone deacetylase inhibitors in solid tumors: a review based on 30 clinical trials. Future Oncol.

[90] Tang S-W, Thomas A, Murai J, Trepel JB, Bates SE, Rajapakse VN, Pommier Y (2018). Overcoming resistance to DNA-targeted agents by epigenetic activation of schlafen 11 ( expression with class I histone deacetylase inhibitors. Clin Cancer Res.

[91] Guan W, Jing Y, Dou L, Wang M, Xiao Y, Yu L (2020). Chidamide in combination with chemotherapy in refractory and relapsed T lymphoblastic lymphoma/leukemia. Leuk Lymphoma.

[92] Mao J, Li S, Zhao H, Zhu Y, Hong M, Zhu H, Qian S, Li J (2018). Effects of chidamide and its combination with decitabine on proliferation and apoptosis of leukemia cell lines. Am J Transl Res.

[93] Guan XW, Wang HQ, Ban WW, Chang Z, Chen HZ, Jia L, Liu FT (2020). Novel HDAC inhibitor Chidamide synergizes with Rituximab to inhibit diffuse large B-cell lymphoma tumour growth by upregulating CD20. Cell Death Dis.

[94] Mu X, Zhao Q, Chen W, Zhao Y, Yan Q, Peng R, Zhu J, Yang C, Lan K, Gu X (2021). IL-37 confers anti-tumor activity by regulation of m6A methylation. Front Oncol.

[95] Selberg S, Blokhina D, Aatonen M, Koivisto P, Siltanen A, Mervaala E, Kankuri E, Karelson M (2019). Discovery of small molecules that activate RNA methylation through cooperative binding to the METTL3-14-WTAP complex active site. Cell Rep.

[96] Ping XL, Sun BF, Wang L, Xiao W, Yang X, Wang WJ, Adhikari S, Shi Y, Lv Y, Chen YS (2014). Mammalian WTAP is a regulatory subunit of the RNA N6-methyladenosine methyltransferase. Cell Res.

[97] Yang F, Yuan W-Q, Li J, Luo Y-Q (2021). Knockdown of METTL14 suppresses the malignant progression of non-small cell lung cancer by reducing Twist expression. Oncol Lett.

[98] Li F, Zhao J, Wang L, Chi Y, Huang X, Liu W (2022). METTL14-mediated miR-30c-1-3p maturation represses the progression of lung cancer via regulation of MARCKSL1 expression. Mol Biotechnol.

[99] Little NA, Hastie ND, Davies RC (2000). Identification of WTAP, a novel Wilms' tumour 1-associating protein. Hum Mol Genet.

[100] Weng L, Qiu K, Gao W, Shi C, Shu F (2020). LncRNA PCGEM1 accelerates non-small cell lung cancer progression via sponging miR-433-3p to upregulate WTAP. BMC Pulm Med.

[101] Sorci M, Ianniello Z, Cruciani S, Larivera S, Ginistrelli LC, Capuano E, Marchioni M, Fazi F, Fatica A (2018). METTL3 regulates WTAP protein homeostasis. Cell Death Dis.

[102] Wei W, Huo B, Shi X (2019). miR-600 inhibits lung cancer via downregulating the expression of METTL3. Cancer Manag Res.

[103] Li X, Li N, Huang L, Xu S, Zheng X, Hamsath A, Zhang M, Dai L, Zhang H, Wong JJ-L (2020). Is hydrogen sulfide a concern during treatment of lung adenocarcinoma with ammonium tetrathiomolybdate?. Front Oncol.

[104] Liu P, Li F, Lin J, Fukumoto T, Nacarelli T, Hao X, Kossenkov AV, Simon MC, Zhang R (2021). m(6)A-independent genome-wide METTL3 and METTL14 redistribution drives the senescence-associated secretory phenotype. Nat Cell Biol.

[105] Wu H, Li F, Zhu R (2021). miR-338-5p inhibits cell growth and migration via inhibition of the METTL3/m6A/c-Myc pathway in lung cancer. Acta Biochim Biophys Sin.

[106] Gong C, Fan Y, Liu J (2021). METTL14 mediated m6A modification to LncRNA ZFAS1/ RAB22A: a novel therapeutic target for atherosclerosis. Int J Cardiol.

[107] Yankova E, Blackaby W, Albertella M, Rak J, De Braekeleer E, Tsagkogeorga G, Pilka ES, Aspris D, Leggate D, Hendrick AG (2021). Small-molecule inhibition of METTL3 as a strategy against myeloid leukaemia. Nature.

